# Multi-component pharmacokinetic study of *prunus mume* fructus extract after oral administration in rats using UPLC-MS/MS

**DOI:** 10.3389/fphar.2022.954692

**Published:** 2022-09-23

**Authors:** Yameng Zhu, Shujie Wei, Xiunan Cao, Songrui Wang, Yanxu Chang, Huizi Ouyang, Jun He

**Affiliations:** State Key Laboratory of Component-based Chinese Medicine, Tianjin University of Traditional Chinese Medicine, Tianjin, China

**Keywords:** Prunus mume fructus extract, prototype components, pharmacokinetic, rat plasma, UPLC-MS/MS

## Abstract

*Prunus mume* fructus (MF) is used in traditional Chinese medicine and food, as it exerts pharmacological effects, such as antibacterial, antioxidant, antitumour, thirst-relieving, and antidiarrheal effects. In the present study, a reliable and sensitive ultra-high performance liquid chromatography/tandem mass spectrometry (UPLC-MS/MS) method was developed and validated for the simultaneous determination of 16 prototype components (L-(-)-malic acid, 3,4-dihydroxybenzaldehyde, protocatechuic acid, vanillic acid, caffeic acid, D-(-)-quinic acid, citric acid, ferulic acid, syringic acid, cryptochlorogenic acid, neochlorogenic acid, chlorogenic acid, amygdalin, maslinic acid, corosolic acid, and rutin) in rat plasma after oral administration of the MF extract. Plasma samples were prepared via protein precipitation using acetonitrile. The 16 components were separated on an ACQUITY UPLC BEH C18 column (2.1 × 100 mm, 1.7 μm) with a gradient mobile phase system of methanol and 0.1% (v/v) formic acid aqueous solution at a flow rate of 0.3 ml/min. All components were quantitated using Agilent Jet Stream electrospray ionisation in negative ion mode. The intra-day and inter-day accuracies ranged from-9.4 to 9.4%, and the precision of the analytes was less than 14.8%. The extraction recovery rate of the analytes ranged from 63.59 to 109.44% and the matrix effects ranged from 49.25 to 109.28%. Stability studies proved that the analytes were stable under the tested conditions, with a relative standard deviation lower than 13.7%. Hence, the developed method was successfully applied to evaluate the pharmacokinetics of 16 components in the MF extract after oral administration in rats using UPLC-MS/MS.

## 1 Introduction

Medicinal plants are a kind of economic plants with special purpose, which have been done since ancient times and have important medical and health functions for human health, and may even be considered the origin of modern medicine. Compounds of plant origin have been and still are an important source of compounds for drugs. It is a fact that all civilizations have developed this form of medicine based on the plants in their own habitat. Therefore, it is of great significance to conduct in-depth research and development of medicinal plants. (Dogan et al., 2014; Saran et al., 2021; Tamer et al., 2021). *Prunus mume* fructus (MF) is derived from the near-mature fruit of the *Rosaceae* plant, *Prunus mume* Sieb. et Zucc., and is used in traditional Chinese medicine and food (Shi et al., 2019; Chinese Pharmacopoeia Commission, 2020). It is widely cultivated in Eastern Asian countries for its flavour and aroma, availability, and nutritional and therapeutic benefits, and is mainly distributed in Korea, Japan, and the Yunnan, Fujian, Sichuan, Anhui, and Guizhou provinces in China (Xu, 1996; Yan et al., 2014). MF is widely used in medications, for astringing the lungs to relieve cough, astringing the intestines, promoting fluid relief, and treating diarrhoea and ascariasis. It is commonly used in herbal medicine to treat chronic cough, prolonged diarrhoea, asthenic fever, and ascaris-induced convulsions (Zhao and Wang, 2010; Chinese Pharmacopoeia Commission, 2020; Xiang et al., 2020; Liu et al., 2022). MF is a popular fruit that is commonly consumed in the form of processed products, such as candy, pickles, beverages, and liquor. In East Asian countries, candied MF is a commonly consumed snack (Imahori et al., 2008; Luo et al., 2018). Chinese people have the habit of drinking sour plum juice made of MF to relieve the summer heat. Nowadays, MF is widely used in health drinks, such as *Imperatae Rhizoma* extracts, and is compounded into drinks in a certain proportion, which confers a unique flavour and greatly improves the nutritional value of the drink. Beverages with nutritional benefits and summer heat-relieving effects can be prepared using MF and mung bean as the main raw materials (Ding, 2012; Fang et al., 2018). Modern pharmacological studies have suggested that MF has biological activities, such as immunity-enhancing, antibacterial, antioxidant, antitumour, antidiarrheal, thirst-relieving, hypouricemic, anti-fatigue, and anti-aging activities (Choi et al., 2007; Yi et al., 2012; Tu et al., 2013; Park et al., 2016; Xing et al., 2018; Zhang et al., 2021). Clinical studies have shown that MF has preventive effects in the treatment of ulcerative colitis, anaemia, bronchial asthma, and diabetes (Kim et al., 2020; Zhu et al., 2022).

Phytochemical studies have shown that MF contains various components, including organic acids, terpenoids, sterols, volatile components, amino acids, carbohydrates, lipids, flavonoids, and alkaloids, of which the most important biologically compounds are organic acids (Turturica et al., 2016; Ou et al., 2020). The organic acids in MF have shown antioxidant, antibacterial, antitumour, and anti-cardiovascular effects (Liu et al., 2019). Terpenoids, sterols, carbohydrates, and alkaloids exert antitumour effects, while amino acids modulate the immune system functions (Wang et al., 2022). Pharmacokinetics is a dynamic process to investigate the absorption, distribution, metabolism and excretion of drugs *in vivo*. The pharmacokinetic characteristics of components in traditional Chinese medicine (TCM) have been used to predict the potential toxicity of drugs, and guide clinical rational drug use (Cheng et al., 2022). The characteristics of the ingredients *in vivo* can be understood by comparing their pharmacokinetic parameters to increase the safety and availability of clinical medications and to aid in determining their appropriate dosage and time of administration (Zhang et al., 2017). Therefore, the pharmacokinetic studies of the main components in MF of great significance to promote its clinical application. However, although MF has various pharmacological effects, its *in vivo* pharmacokinetics have not yet been elucidated.

In this study, a reliable and sensitive ultra-high performance liquid chromatography/tandem mass spectrometry (UPLC-MS/MS) method was established and validated for the simultaneous determination of 16 components (L-(-)-malic acid, 3,4-dihydroxybenzaldehyde, protocatechuic acid, vanillic acid, caffeic acid, D-(-)-quinic acid, citric acid, ferulic acid, syringic acid, cryptochlorogenic acid, neochlorogenic acid, chlorogenic acid, amygdalin, maslinic acid, corosolic acid, and rutin) in rat plasma. The method was then applied to the pharmacokinetic study of the compounds in rats after oral administration of the MF extract to obtain valuable information for the development and clinical application of MF.

## 2 Materials and methods

### 2.1 Chemicals and reagents

**FIGURE 1:**
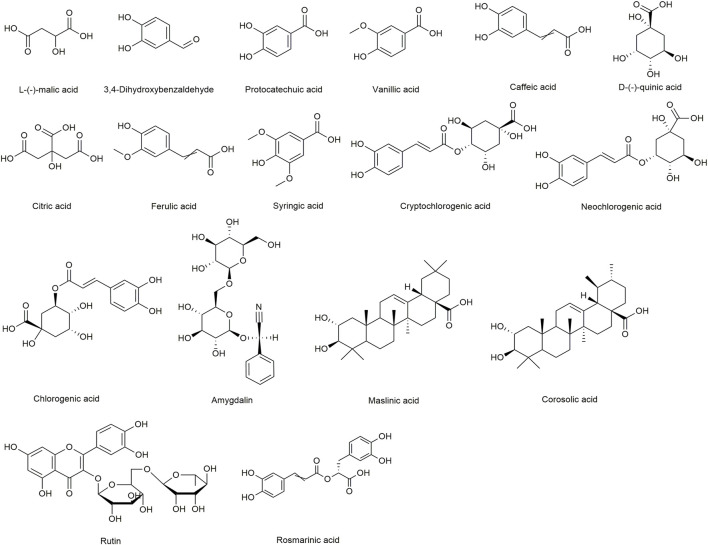
The chemical structures of the 16 analytes and IS.

Standards, L-(-)-malic acid, 3,4-Dihydroxybenzaldehyde, protocatechuic acid, vanillic acid, caffeic acid, D-(-)-quinic acid, citric acid, ferulic acid, syringic acid, cryptochlorogenic acid, neochlorogenic acid, chlorogenic acid, amygdalin, maslinic acid, corosolic acid, rutin, and rosmarinic acid (internal standard [IS], purity ≥98%) were purchased from Chengdu Desite Bio-Technology Co., Ltd. (Chengdu, China). Amygdalin was obtained from the National Institutes for Food and Drug Control (Beijing, China). The chemical structures of the 16 analytes and IS are shown in Figure 1. Methanol and acetonitrile (chromatographic grade) were supplied by Fisher Scientific (Fair Lawn, NJ, USA). Formic acid (chromatographic grade) was obtained from ROE (St. Louis, MO, USA). Demineralised water was obtained using a Milli-Q water purification system (Millipore, Milford, MA, USA). MF, the near-mature fruit of the Rosaceae plant, was collected from May to July 2020 in Sichuan province in China. The raw materials were carefully identified by Professor Jun He from Tianjin University of Traditional Chinese Medicine (TUTCM), China.

### 2.2 Chromatography and mass spectrometry

The samples were analyzed and monitored using a UPLC–MS/MS system. It was mainly composed of an Agilent 1,290 ultra-high-performance liquid chromatography system (Agilent, USA) and an Agilent 6,470 series triple quadrupole mass spectrometer (Agilent, USA) equipped with an electrospray ionisation source.

Separation was performed on an ACQUITY UPLC BEH C18 (2.1 × 100 mm, 1.7 µm), and the column temperature was maintained at 25°C. The mobile phase consisted of solvent A (0.1% [v/v] formic acid in water) and solvent B (methanol). The gradient elution was set as follows: 10–25% B at 0–1 min; 25–30% B at 1–3.5 min; 30–40% B at 3.5–4.5 min; 40–83% B at 4.5–5.0 min; 83–83% B at 5.0–11.5 min; 83–100% B at 11.5–12.0 min; and 100–100% B at 12.0–13.0 min. The flow rate was set at 0.3 ml/min and the injection volume was 2 μL.

Multiple reaction monitoring (MRM) was used for quantitative analysis in both the positive and negative ionisation modes. The mass spectrometric parameters were set as follows: drying gas (N_2_) temperature, 300°C; gas (N_2_) flow rate, 7 L/min; atomiser pressure, 35 psi; sheath gas temperature, 350°C; sheath gas flow, 11 L/min; capillary voltage, 3500 V. The specific mass spectrometric parameters of the precursor ion, product ion, fragmentor, collision energy, and ion mode of the 16 components and the IS are listed in Supplementary Table S1.

### 2.3 *Prunus mume* fructus extract preparation

*Prunus mume* fructus extract was prepared as follows: A total of 600.0 g of MF was accurately weighed and extracted twice under heat reflux using eight times the amount of 50% ethanol (v/v) for 2 h each time. The extraction solutions were then filtered and mixed. The mixed solution was concentrated via evaporation under reduced pressure. Finally, the dried MF extract was pulverised into a fine powder and stored in a desiccator until analysis. The contents of L-(-)-malic acid, 3,4-dihydroxybenzaldehyde, protocatechuic acid, vanillic acid, caffeic acid, D-(-)-quinic acid, citric acid, ferulic acid, syringic acid, cryptochlorogenic acid, neochlorogenic acid, chlorogenic acid, amygdalin, maslinic acid, corosolic acid, and rutin in the MF extract are listed in Supplementary Table S2.

### 2.4 Preparation of standard solutions, calibration standards and quality control samples

L-(-)-Malic acid, citric acid, D-(-)-quinic acid, and protocatechuic acid were weighed separately and dissolved in deionised water as standard stock solution (1 mg/ml). Then, 3,4-dihydroxybenzaldehyde, vanillic acid, caffeic acid, ferulic acid, syringic acid, cryptochlorogenic acid, neochlorogenic acid, chlorogenic acid, amygdalin, maslinic acid, corosolic acid, rutin, and rosmarinic acid (IS) were weighed separately and diluted with methanol as a standard stock solution (1 mg/ml).

The calibration solutions were prepared by adding appropriate volumes of the mixture working solution, 20 μL of IS, and 10 μL of formic acid into 100 μL rat plasma, resulting in the following concentrations: 200, 400, 800, 1,600, 4,000, 10000, 20000, 50000, and 100000 ng/ml for citric acid; 20, 40, 80, 160, 400, 1,000, 2000, 5,000, and 10000 ng/ml for D-(-)-quinic and L-(-)-malic acid; 2, 4, 8, 16, 40, 100, 200, 500, and 1,000 ng/ml for vanillic acid, protocatechuic acid, caffeic acid, cryptochlorogenic acid, neochlorogenic acid, chlorogenic acid, and amygdalin; 1, 2, 4, 8, 20, 50, 100, 250, and 500 ng/ml for syringic, corosolic, and maslinic acids; 0.5, 1, 2, 4, 10, 25, 50, 125, and 250 ng/ml for ferulic acid, rutin, and 3,4-dihydroxybenzaldehyde. Quality control (QC) samples at three concentrations (low, medium, and high) were prepared in the same manner. All solutions were stored at 4°C until analysis.

### 2.5 Plasma sample preparation

Plasma (100 μL) was mixed with 20 μL of methanol, 20 μL of IS (1,000 ng/ml), and 10 μL of formic acid, and then vortexed for 1 min. The mixture was extracted with 1 ml acetonitrile by vortexing for 5 min at room temperature. After centrifugation at 14,000 rpm for 10 min, the supernatant was collected in a clean tube and evaporated to dryness under a nitrogen stream. The residue was reconstituted in 100 μL of 50% methanol, vortexed for 5 min, and centrifuged at 14,000 rpm for 10 min. Finally, 10 μL of the supernatant was injected into the UPLC-MS/MS system for analysis.

### 2.6 Method validation

The UPLC-MS/MS method for the determination of 16 components in rat plasma was validated according to the US FDA Bioanalytical Method Validation Guidance (Ma et al., 2021; Liu et al., 2022), including specificity, linearity, lower limit of quantification (LLOQ), accuracy, precision, extraction recovery, matrix effect and stability.

#### 2.6.1 Specificity

Specificity was assessed by comparing the chromatograms of the blank plasma samples, corresponding blank plasma samples mixed with the analytes and IS, and plasma samples collected after oral administration of the MF extract in rats. However, blank plasma samples without analytes could not be obtained for L-(-)-malic and citric acids, as they are endogenous compounds in MF. In this study, the chromatograms of the sample without the reference substance solution were compared with the standard solution of a certain concentration, and the specificity was determined based on whether the retention times of L-(-)-malic and citric acids in each ion monitoring channel were consistent.

#### 2.6.2 Linearity and LLOQ

Calibration curves were obtained by plotting the relationship between the peak area ratios of each analyte to IS versus the concentration of the corresponding analyte. A linear regression equation with a weighting coefficient of 1/x^2^ was used to describe the regression relationship. LLOQ was assessed according to baseline noise, with a signal-to-noise ratio of approximately 10.

#### 2.6.3 Precision and accuracy

Precision and accuracy were evaluated by analysing six replicates of QC samples at low, medium, and high concentrations (400, 4,000, and 80000 ng/ml for citric acid; 40, 400, and 8,000 ng/ml for D-(-)-quinic and L-(-)-malic acids; 4, 40, and 800 ng/ml for vanillic acid, protocatechuic acid, caffeic acid, cryptochlorogenic acid, neochlorogenic acid, chlorogenic acid, and amygdalin; 2, 20, and 400 ng/ml for syringic, corosolic, and maslinic acids; 1, 10, and 200 ng/ml for ferulic acid, rutin and 3,4-dihydroxybenzaldehyde) within 1 day or on three consecutive days. Accuracy was determined using relative error (RE%), while intra- and inter-day precisions were determined using relative standard deviation (RSD).

#### 2.6.4 Extraction recovery and matrix effect

The extraction recovery rates and matrix effects were investigated using six replicates at three concentrations. The extraction recovery rates of analytes were measured by the peak areas of analytes with three concentrations and the peak areas of the post-extraction mixed samples. The matrix effects were evaluated by calculating the ratio of the peak area of the analytes in the post-extracted mixed samples to those of the standard solutions. However, L-(-)-malic and citric acids are endogenous substances in MF; therefore, it is necessary to exclude their effects on the extraction recovery rate and matrix effects in the blank plasma (He, 2013). The extraction recovery rate (Equation 1) and matrix effects (Equation 2) of L-(-)-malic and citric acids were calculated as follows:

$$Extraction\;recovery\;\left(\%\right)=\frac{A–C}{B–C}\times 100\%\;$$ (1)

$$Matrix\;effect\;\left(\%\right)=\frac{A–C}{D}\times 100\%\;$$ (2)

where A is the peak area of L-(-)-malic and citric acids in the pre-extracted and mixed samples, B is the peak area of L-(-)-malic and citric acids in the post-extracted and mixed samples, C is the peak area of L-(-)-malic and citric acids in the blank plasma, and D is the peak area of the standard solution.

#### 2.6.5 Stability

The stability of the analytes in plasma samples was evaluated by analysing the QC samples at three concentrations under different conditions: stored in an auto-sampler for 12 h, at room temperature for 4 h, under three freeze-thaw cycles, and stored at -80°C for 7 days.

### 2.7 Pharmacokinetic study

Male Sprague-Dawley rats (220 ± 10 g) were obtained from Beijing HUAFUKANG Bioscience Co., Inc. (Beijing, China). Six male rats were used for this experiment and were allowed free access to water and fasted for 12 h prior to the study. An aqueous solution of 0.5% CMC-Na was used to dissolve the MF extract to a concentration of 0.5 g/ml. The rats were orally administered a dose of 5.0 g/kg suspension, and approximately 300 μL blood samples were collected from the fundus venous plexus of rats before and after oral administration at 0, 0.03, 0.083, 0.17, 0.25, 0.5, 0.75, 1, 2, 4, 6, 8, 10, 12, 24, 36, and 48 h. After centrifugation at 7,000 rpm for 10 min, the collected plasma was transferred into clean tubes and frozen at –80°C until analysis.

### 2.8 Data analysis

Data are presented as the mean ± standard deviation. The plasma concentrations of the 16 analytes were quantitatively calculated using the MassHunter Workstation software (version B.09.00, Agilent, USA). The pharmacokinetic results were processed using the DAS Software (DAS 3.0; Medical College of Wannan, China) to evaluate the exact pharmacokinetic parameters, including time for concentration maximum (T_max_), plasma half-life (T_1/2_), concentration maximum (C_max_), area-under-the-curve (AUC) and mean residence time (MRT).

## 3 Result and discussion

### 3.1 Optimization of chromatography and MS

**FIGURE 2:**
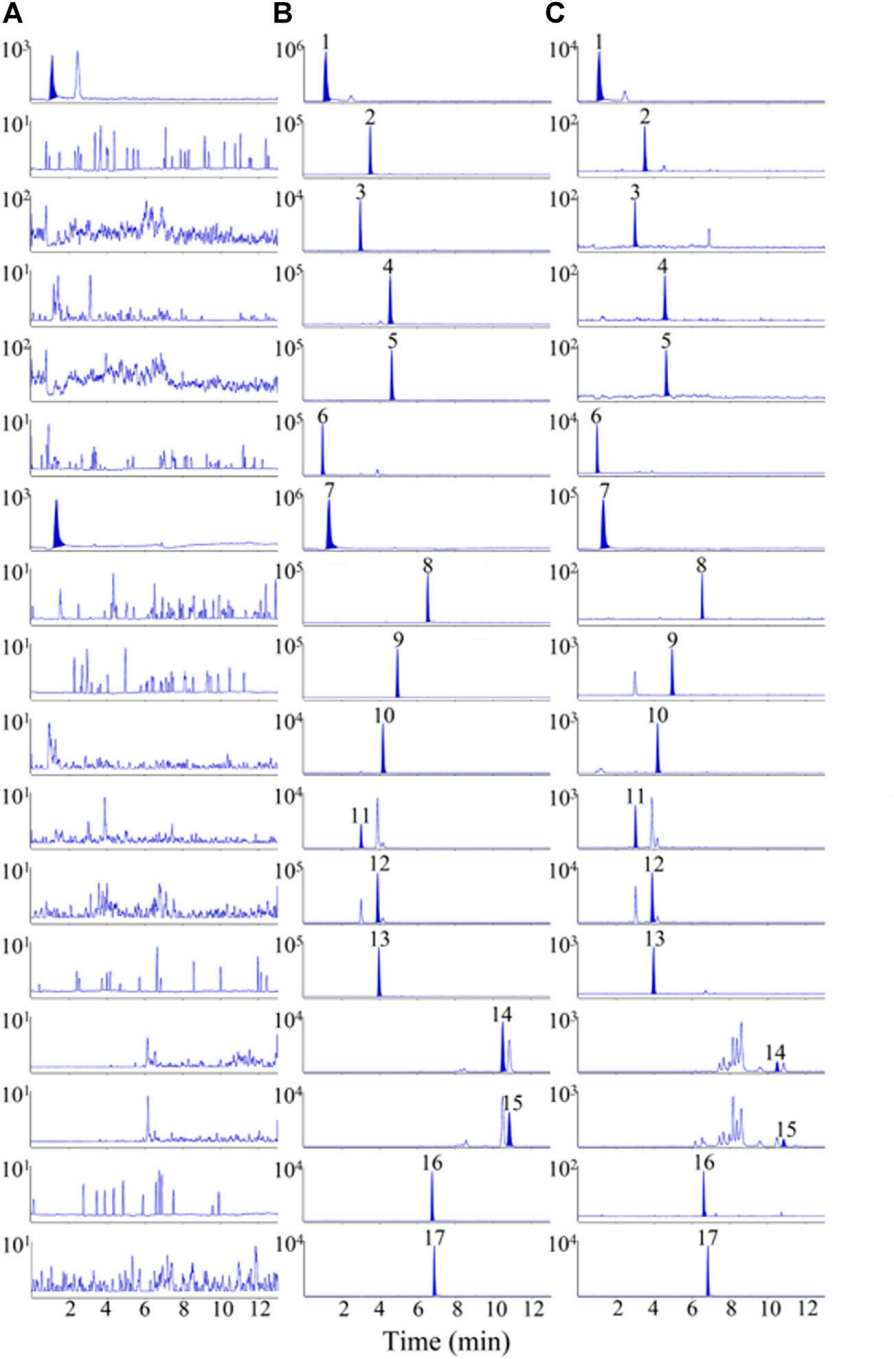
Representative multiple reaction monitoring (MRM) chromatograms of 16 analytes and IS in rat plasma samples. **(A)** blank plasma sample, **(B)** blank plasma spiked with 16 analytes and IS, **(C)** plasma samples after oral administration of MF extract. 1. L-(-)-malic acid, 2.3,4-Dihydroxybenzaldehyde, 3. Protocatechuic acid, 4. Vanillic acid, 5. caffeic acid, 6. D-(-)-quinic acid, 7. citric acid, 8. ferulic acid, 9. syringic acid, 10. cryptochlorogenic acid, 11. neochlorogenic acid, 12. chlorogenic acid, 13. amygdalin, 14. maslinic acid, 15. corosolic acid, 16. rutin, 17. rosmarinic acid (IS).

To achieve better separation of the 16 analytes, the effects of the Waters ACQUITY UPLC BEH C18 (2.1 × 100 mm, 1.7 µm) and Waters ACQUITY UPLC HSS T3 (2.1 × 100 mm, 1.8 µm) columns on chromatographic peaks and elution time were investigated. The results indicated that Waters ACQUITY UPLC BEH C18 (2.1 × 100 mm, 1.7 µm) column had a better separation effect on the 16 analytes, with a shorter elution time. Additionally, various mobile phases, including 0.1% (v/v) formic acid/water–acetonitrile, 0.1% (v/v) formic acid/water–methanol, water–methanol, and water–acetonitrile, were studied to optimise the separation of the 16 analytes. The results showed that 0.1% (v/v) formic acid/water–methanol had a better effect on the separation and peak shape of the analytes, as shown in Figure 2. Sixteen analytes and IS were eluted within 12 min, and no interference peaks were observed.

The main MS parameters were optimised to obtain a better response. Compared with the positive ion mode, the negative ion mode showed a greater signal intensity. Therefore, the 16 analytes were quantitated using Agilent Jet Stream electrospray ionisation in the negative ion mode. In addition, the capillary temperature, atomiser pressure, drying gas flow, fragmentor, and collision energy were optimised to obtain the most appropriate electrospray ionisation parameters, as described in Section 2.2.

### 3.2 Sample preparation

In this study, three extraction methods, namely the methanol-precipitated protein, acetonitrile-precipitated protein, and ethyl acetate liquid–liquid extraction, were investigated to find a suitable method for the preparation of plasma samples. The results demonstrated that the acetonitrile-precipitated protein method had better extraction recovery for the 16 analytes. To solve the problem of the low extraction recovery of chlorogenic, cryptochlorogenic, neochlorogenic, and protocatechuic acids, two different acids (formic and acetic acids) were added to acetonitrile and compared. The results showed that the addition of formic acid had a better effect on the extraction recovery of the 16 analytes; thus, the protein precipitation method using acetonitrile containing formic acid was used for the sample preparation of 16 analytes. Furthermore, the effects of methanol, acetonitrile, 50% methanol, and methanol–acetonitrile (v/v = 1:1) reconstitution solvents were investigated, and the results showed that 50% methanol had the best redissolution effect.

### 3.3 Method validation

#### 3.3.1 Specificity

MRM chromatograms of the blank plasma sample (A), blank plasma sample mixed with the 16 analytes and IS (B), and plasma samples collected after oral administration of the MF extract (C) are shown in Figure 2. The chromatograms of the samples without the reference substance solution were compared with the standard solution of specific concentration, as shown in Figure 2, where L-(-)-malic and citric acids were endogenous substances, and no obvious interfering peaks were observed in the retention period of the other 14 analytes.

#### 3.3.2 Linearity and LLOQ

The regression equations, linear ranges, correlation coefficients, and LLOQ values of the 16 analytes are listed in Table 1. The calibration profiles of the 16 analytes showed good linearity within the corresponding concentration ranges (*r* ^2^ > 0.994). The LLOQ values of L-(-)-malic acid, 3,4-dihydroxybenzaldehyde, protocatechuic acid, vanillic acid, caffeic acid, D-(-)-quinic acid, citric acid, ferulic acid, syringic acid, cryptochlorogenic acid, neochlorogenic acid, chlorogenic acid, amygdalin, maslinic acid, corosolic acid, and rutin were 20.0, 0.5, 2.0, 2.0, 2.0, 20.0, 40.0, 0.5, 1.0, 0.06, 0.1, 0.2, 1.0, 1.0, 1.0, and 0.2 ng/ml, respectively.

**TABLE 1:** Calibration profiles, linear ranges, correlation coefficients and LLOQ of the 16 analytes.

Compound	Calibration curve	Correlation coefficients (*r* ^2^)	Linear range (ng/ml)	LLOQ (ng/ml)
L-(-)-malic acid	Y = 0.4212X+0.5209	0.9957	20.0–10000.0	20.0
3,4-Dihydroxybenzaldehyde	Y = 1.7801X-8.4861	0.9987	0.5–250.0	0.5
Protocatechuic acid	Y = 3.5217X-0.0019	0.9983	2.0–1,000.0	2.0
Vanillic acid	Y = 0.1878X-1.5254	0.9972	2.0–1,000.0	2.0
Caffeic acid	Y = 7.3240X-0.0036	0.9986	2.0–1,000.0	2.0
D-(-)-quinic acid	Y = 0.0587X-6.9144	0.9956	20.0–10000.0	20.0
Citric acid	Y = 0.3677X+6.4005	0.9952	200.0–100000.0	40.0
Ferulic acid	Y = 0.9976X+3.1040	0.9958	0.5–250.0	0.5
Syringic acid	Y = 0.6823X+3.7770	0.9973	1.0–500.0	1.0
Cryptochlorogenic acid	Y = 2.7567X-0.0030	0.9972	2.0–1,000.0	0.06
Neochlorogenic acid	Y = 6.4283X-0.0050	0.9969	2.0–1,000.0	0.1
Chlorogenic acid	Y = 14.4709X-0.0156	0.9976	2.0–1,000.0	0.2
Amygdalin	Y = 0.5475X-6.5412	0.9947	2.0–1,000.0	1.0
Maslinic acid	Y = 11.5563X-0.0113	0.9971	1.0–500.0	1.0
Corosolic acid	Y = 8.9094X+0.0315	0.9960	1.0–500.0	1.0
Rutin	Y = 3.5615X-6.9727	0.9985	0.5–250.0	0.2

#### 3.3.3 Precision and accuracy

The accuracy and intra- and inter-day precision of QC samples at low, medium, and high concentrations are listed in Table 2. The intra- and inter-day RSD values were less than 14.8%, the intra-day RE ranged from –9.4–9.9%, while the inter-day RE ranged from –8.5–9.4%. These results suggest that this method has acceptable limits of precision and accuracy.

**TABLE 2:** Precision and accuracy of 16 analytes in rat plasma (*n* = 6).

Compound	Spiked concent-ration (ng/ml)	Intra-day			Inter-day		
		Measured (ng/ml)	RE (%)	RSD (%)	Measured (ng/ml)	RE (%)	RSD (%)
L-(-)-malic acid	40	42.60 ± 2.77	6.5	6.5	41.79 ± 2.66	4.5	6.4
	400	403.32 ± 41.18	0.8	10.2	408.71 ± 47.49	2.2	11.6
	8,000	8,298.08 ± 241.90	3.7	2.9	8,485.26 ± 478.91	6.1	5.6
3,4-Dihydroxybenzaldehyde	1	0.91 ± 0.05	−9.4	5.5	0.95 ± 0.03	−5.1	3.4
	10	9.33 ± 0.64	−6.7	6.9	9.65 ± 0.59	−3.5	6.1
	200	186.79 ± 9.50	−6.6	5.1	185.27 ± 9.11	−7.4	4.9
Protocatechuic acid	4	4.38 ± 0.29	9.5	6.7	4.35 ± 0.12	8.8	2.8
	40	39.51 ± 1.63	−1.2	4.1	40.55 ± 1.96	1.4	4.8
	800	812.01 ± 16.24	1.5	2.0	808.99 ± 6.46	1.1	0.8
Vanillic acid	4	4.13 ± 0.24	3.3	5.8	4.09 ± 0.31	2.4	7.5
	40	40.82 ± 3.13	2.1	7.7	39.03 ± 1.96	−2.4	5.0
	800	827.29 ± 26.5	3.4	3.2	811.25 ± 24.99	1.4	3.1
Caffeic acid	4	4.04 ± 0.39	1.0	9.6	4.09 ± 0.23	2.2	5.6
	40	41.55 ± 6.14	3.9	14.8	42.58 ± 2.87	6.5	6.8
	800	805.06 ± 23.25	0.6	2.9	811.21 ± 25.92	1.4	3.2
D-(-)-quinic acid	40	42.83 ± 3.39	7.1	7.9	41.87 ± 2.62	4.7	6.3
	400	400.58 ± 9.89	0.1	2.5	393.16 ± 6.42	−1.7	1.6
	8,000	7,814.57 ± 393.54	−2.3	5.0	8,232.43 ± 289.12	2.9	3.5
Citric acid	400	392.35 ± 24.29	−1.9	6.2	403.99 ± 40.89	1.0	10.1
	4,000	4,148.58 ± 284.96	3.7	6.9	4,110.18 ± 163.12	2.8	4.0
	80000	79004.06 ± 1,081.18	−1.2	1.4	79443.42 ± 866.91	-0.7	1.1
Ferulic acid	1	1.02 ± 0.09	1.6	8.9	1.09 ± 0.09	9.4	8.1
	10	10.92 ± 0.65	9.2	6.0	10.66 ± 0.68	6.6	6.4
	200	200.74 ± 5.93	0.4	3.0	201.97 ± 7.36	1.0	3.6
Syringic acid	2	2.20 ± 0.12	9.9	5.3	1.97 ± 0.17	−1.6	8.6
	20	19.86 ± 1.38	-0.7	7.0	20.15 ± 1.07	0.8	5.3
	400	416.66 ± 18.54	4.2	4.5	417.07 ± 10.37	4.3	2.5
Cryptochlorogenic acid	4	4.34 ± 0.40	8.5	9.2	4.23 ± 0.33	5.8	7.9
	40	40.71 ± 3.13	1.8	7.7	39.73 ± 3.06	−0.7	7.7
	800	778.98 ± 22.80	-2.6	2.9	808.44 ± 37.23	1.1	4.6
Neochlorogenic acid	4	4.21 ± 0.40	5.3	9.4	4.11 ± 0.14	2.7	3.4
	40	39.38 ± 0.73	−1.5	1.9	40.88 ± 3.49	2.2	8.5
	800	794.74 ± 19.23	−0.7	2.4	805.56 ± 17.04	0.7	2.1
Chlorogenic acid	4	4.18 ± 0.25	4.5	6.0	4.08 ± 0.44	2.1	10.7
	40	38.08 ± 1.48	-4.8	3.9	40.98 ± 1.97	2.5	4.8
	800	805.30 ± 18.09	0.7	2.3	810.87 ± 6.18	1.4	0.8
Amygdalin	4	4.18 ± 0.20	4.6	4.8	4.24 ± 0.38	6.1	9.0
	40	38.97 ± 1.19	−2.6	3.1	40.93 ± 1.91	2.3	4.7
	800	787.23 ± 24.20	−1.6	3.1	821.56 ± 38.30	2.7	4.7
Maslinic acid	2	2.04 ± 0.11	2.1	5.2	1.93 ± 0.09	−3.5	4.9
	20	18.51 ± 1.21	−7.5	6.6	18.29 ± 2.27	−8.5	12.4
	400	371.59 ± 22.98	−7.1	6.2	394.93 ± 12.94	−1.3	3.3
Corosolic acid	2	1.96 ± 0.15	−2.0	7.5	2.07 ± 0.18	3.3	8.9
	20	19.08 ± 0.83	−4.6	4.4	19.01 ± 1.73	−4.9	9.1
	400	379.53 ± 10.99	−5.1	2.9	406.96 ± 16.16	1.7	4.0
Rutin	1	1.05 ± 0.08	4.5	7.4	0.97 ± 0.06	−2.9	6.6
	10	9.33 ± 0.18	−6.7	2.0	9.83 ± 0.68	−1.7	6.9
	200	188.40 ± 7.88	−5.8	4.2	211.95 ± 20.20	6.0	9.5

#### 3.3.4 Extraction recovery and matrix effect

The extraction recovery rates and matrix effects of the analytes at low, medium, and high concentrations are summarised in Table 3. The extraction recovery rates of all analytes ranged from 80.86 to 109.44% (RSD <10.3%). The matrix effects of L-(-)-malic and citric acids at different concentrations ranged from 49.25 to 69.04% (RSD <10.7%). However, the matrix effects remained similar at different concentrations (low, medium, and high), and the consistency was good; thus, the matrix effects of L-(-)-malic and citric acids did not affect the experimental measurements. Moreover, the matrix effects on other analytes ranged from 70.02 to 109.28% (RSD <11.2%). These results demonstrate that the extraction recovery rates and matrix effects of this method are precise and acceptable.

**TABLE 3:** Extraction recovery and matrix effects of 16 analytes in rat plasma (*n* = 6).

Compound	Spiked concentration (ng/ml)	Extraction recovery (%)	RSD (%)	Matrix effect (%)	RSD (%)
L-(-)-malic acid	40	89.09 ± 4.72	5.3	56.13 ± 5.56	9.9
	400	88.83 ± 3.06	3.5	55.17 ± 2.26	4.1
	8,000	87.63 ± 5.07	5.8	49.25 ± 1.48	3.0
3,4-Dihydroxybenzaldehyde	1	90.11 ± 5.28	5.9	91.73 ± 7.88	8.6
	10	82.65 ± 2.31	2.8	91.27 ± 3.85	4.2
	200	90.56 ± 2.77	3.1	101.14 ± 2.88	2.9
Protocatechuic acid	4	87.39 ± 8.15	9.3	75.69 ± 8.10	10.7
	40	89.07 ± 7.49	8.4	79.69 ± 5.13	6.4
	800	88.39 ± 5.00	5.7	82.56 ± 3.88	4.7
Vanillic acid	4	89.44 ± 8.34	9.3	97.48 ± 6.50	6.7
	40	92.14 ± 9.07	9.8	87.95 ± 4.37	5.0
	800	92.16 ± 6.26	6.8	93.81 ± 4.74	5.1
Caffeic acid	4	95.04 ± 1.41	1.5	101.25 ± 6.64	6.6
	40	92.47 ± 5.53	6.0	87.48 ± 7.86	9.0
	800	90.07 ± 4.52	5.0	97.56 ± 4.77	4.9
D-(-)-quinic acid	40	85.25 ± 7.58	8.9	70.93 ± 7.91	11.2
	400	80.86 ± 8.32	10.3	71.82 ± 6.5	9.1
	8,000	85.93 ± 3.29	3.8	70.02 ± 2.04	2.9
Citric acid	400	63.59 ± 5.99	9.4	65.53 ± 6.99	10.7
	4,000	67.36 ± 3.09	4.6	69.04 ± 2.84	4.1
	80000	66.43 ± 2.03	3.1	62.13 ± 1.12	1.8
Ferulic acid	1	95.67 ± 3.33	3.5	109.28 ± 11.90	10.9
	10	88.79 ± 1.70	1.9	90.87 ± 8.18	9.0
	200	89.06 ± 4.29	4.8	91.73 ± 3.43	3.7
Syringic acid	2	109.44 ± 7.26	6.6	94.93 ± 6.85	7.2
	20	91.43 ± 7.21	7.9	95.51 ± 7.24	7.6
	400	88.78 ± 4.28	4.8	83.94 ± 4.05	4.8
Cryptochlorogenic acid	4	93.37 ± 4.77	5.1	99.51 ± 4.96	5.0
	40	80.60 ± 3.09	3.8	90.19 ± 2.06	2.3
	800	89.21 ± 2.62	2.9	96.69 ± 4.29	4.4
Neochlorogenic acid	4	84.36 ± 7.84	9.3	83.99 ± 7.71	9.2
	40	90.81 ± 9.12	10.1	85.78 ± 7.44	8.7
	800	89.82 ± 3.35	3.7	78.97 ± 3.57	4.5
Chlorogenic acid	4	92.44 ± 8.41	9.1	87.24 ± 7.49	8.6
	40	89.05 ± 2.32	2.6	85.86 ± 2.12	2.5
	800	86.23 ± 3.21	3.7	93.18 ± 2.56	2.8
Amygdalin	4	92.24 ± 8.47	9.2	95.16 ± 7.28	7.7
	40	96.05 ± 8.26	8.6	89.30 ± 4.92	5.5
	800	88.94 ± 2.54	2.9	82.67 ± 3.18	3.9
Maslinic acid	2	90.15 ± 5.32	5.9	85.64 ± 7.21	8.4
	20	90.32 ± 4.48	5.0	90.92 ± 6.70	7.4
	400	81.73 ± 3.60	4.4	92.36 ± 3.47	3.8
Corosolic acid	2	83.69 ± 3.65	4.4	107.62 ± 8.13	7.6
	20	92.17 ± 5.09	5.5	104.71 ± 5.31	5.1
	400	86.06 ± 3.57	4.2	87.84 ± 2.45	2.8
Rutin	1	89.72 ± 4.27	4.8	87.15 ± 8.16	9.4
	10	86.42 ± 4.17	4.8	89.31 ± 6.89	7.7
	200	86.05 ± 6.11	7.1	95.66 ± 2.53	2.6

#### 3.3.5 Stability

The stability of the QC samples under different conditions are presented in Table 4. The analytes were found to be stable for 4 h at room temperature, for 12 h in an autosampler after preparation, for three freeze-thaw cycles, and for 7 days at -80°C. The RSD values of all tested analytes were below 13.7%, suggesting that all analytes were reasonably stable under different conditions and that the developed UPLC-MS/MS method could be used to determine the 16 analytes in rat plasma.

**TABLE 4:** Stability of 16 analytes in rat plasma (*n* = 6).

Compound	Spiked concentration (ng/ml)	Room temperature For 4 h		Autosampler for 12 h		Three freeze-thaw cycles		-80°C for 7 days	
		Measured (ng/ml)	RSD (%)	Measured (ng/ml)	RSD (%)	Measured (ng/ml)	RSD (%)	Measured (ng/ml)	RSD (%)
L-(-)-malic acid	40	42.19 ± 3.93	9.3	42.61 ± 3.21	7.5	40.44 ± 3.03	7.5	43.18 ± 2.74	6.4
	400	396.66 ± 10.80	2.7	415.57 ± 19.19	4.6	419.22 ± 16.78	4.0	431.91 ± 21.16	4.9
	8,000	7,955.67 ± 96.54	1.2	8,449.27 ± 221.29	2.6	7,893.43 ± 327.30	4.2	8,252.44 ± 264.57	3.2
3,4-Dihydroxybenzaldehyde	1	1.07 ± 0.09	8.8	0.95 ± 0.06	6.2	0.98 ± 0.04	4.3	0.99 ± 0.08	8.2
	10	8.64 ± 2.06	2.4	9.32 ± 0.89	9.5	9.68 ± 0.90	9.3	9.72 ± 0.32	3.3
	200	207.04 ± 3.39	1.6	192.17 ± 11.28	5.9	217.39 ± 8.78	4.0	203.58 ± 16.82	8.3
Protocatechuic acid	4	4.11 ± 0.12	3.0	4.19 ± 0.12	2.9	4.22 ± 0.34	8.2	4.39 ± 0.43	9.7
	40	39.94 ± 0.89	0.3	40.11 ± 1.92	4.8	38.89 ± 3.78	9.7	36.44 ± 3.81	10.5
	800	802.20 ± 3.79	0.5	809.86 ± 17.43	2.2	776.36 ± 27.39	3.5	819.02 ± 22.44	2.7
Vanillic acid	4	4.08 ± 0.49	12.1	4.11 ± 0.46	11.1	4.05 ± 0.15	3.7	4.04 ± 0.13	3.2
	40	37.53 ± 2.81	7.5	40.35 ± 4.48	11.1	39.42 ± 1.47	3.7	36.10 ± 4.94	13.7
	800	771.75 ± 47.24	6.1	803.29 ± 16.28	2.0	766.31 ± 55.15	7.2	778.36 ± 48.13	6.2
Caffeic acid	4	4.14 ± 0.17	4.1	3.99 ± 0.29	7.2	3.97 ± 0.09	2.2	4.01 ± 0.11	2.7
	40	38.23 ± 0.52	1.4	39.94 ± 1.10	2.8	39.50 ± 3.37	8.5	38.92 ± 1.21	3.1
	800	790.38 ± 14.93	1.9	796.14 ± 10.44	1.3	771.72 ± 46.16	6.0	763.35 ± 73.38	9.6
D-(-)-quinic acid	40	41.12 ± 2.09	5.1	42.48 ± 3.71	8.7	40.96 ± 3.50	8.5	39.98 ± 4.12	10.3
	400	417.06 ± 22.55	5.4	415.38 ± 32.71	7.9	405.09 ± 14.67	3.6	377.03 ± 22.82	6.1
	8,000	8,240.54 ± 134.24	1.6	8,278.60 ± 400.24	4.8	8,194.03 ± 253.85	3.1	7,947.60 ± 217.60	2.7
Citric acid	400	402.73 ± 28.85	7.2	408.83 ± 16.28	4.0	410.97 ± 15.49	3.8	416.56 ± 20.49	4.9
	4,000	3998.72 ± 222.26	5.6	4,189.40 ± 106.11	2.5	4,055.00 ± 164.95	4.1	4,035.84 ± 205.39	5.1
	80000	82339.24 ± 3601.54	4.4	79639.90 ± 3375.19	4.2	76762.25 ± 6,277.19	8.2	79007.02 ± 4,141.17	5.2
Ferulic acid	1	1.02 ± 0.11	10.7	1.04 ± 0.06	6.0	1.03 ± 0.05	4.6	0.99 ± 0.08	7.6
	10	9.44 ± 0.92	9.7	10.87 ± 1.12	10.3	10.25 ± 0.33	3.2	9.66 ± 0.83	8.6
	200	187.34 ± 7.03	3.8	211.00 ± 7.93	3.8	190.39 ± 7.49	3.9	194.48 ± 12.71	6.5
Syringic acid	2	1.98 ± 0.13	6.8	2.09 ± 0.16	7.5	2.12 ± 0.20	9.6	2.07 ± 0.16	7.5
	20	19.24 ± 0.73	3.8	21.48 ± 0.52	2.4	19.79 ± 1.82	9.2	19.30 ± 1.19	6.2
	400	361.58 ± 38.52	10.7	419.30 ± 12.25	2.9	383.29 ± 18.87	4.9	381.90 ± 34.04	8.9
Cryptochloro-genic acid	4	4.00 ± 0.17	4.3	4.35 ± 0.21	4.9	4.20 ± 0.12	2.8	4.06 ± 0.27	6.6
	40	38.42 ± 1.23	3.2	39.33 ± 1.72	4.4	40.16 ± 1.01	2.5	40.16 ± 2.31	5.8
	800	801.21 ± 12.79	1.6	809.21 ± 16.15	2.0	794.39 ± 15.58	2.0	791.75 ± 27.72	3.5
Neochloro-genic acid	4	4.03 ± 0.12	3.1	4.29 ± 0.33	7.8	4.39 ± 0.54	12.3	4.29 ± 0.29	6.9
	40	38.43 ± 1.06	2.8	39.30 ± 1.29	3.3	39.64 ± 1.87	4.7	38.81 ± 1.15	3.0
	800	802.24 ± 23.26	2.9	800.32 ± 18.84	2.4	807.78 ± 28.15	3.5	809.12 ± 22.41	2.8
Chlorogenic acid	4	4.08 ± 0.06	1.4	4.40 ± 0.37	8.5	4.28 ± 0.18	4.2	4.19 ± 0.20	4.8
	40	38.54 ± 0.80	2.1	38.96 ± 0.66	1.7	37.46 ± 2.41	6.4	39.26 ± 0.76	1.9
	800	801.19 ± 6.82	0.9	813.31 ± 17.19	2.1	815.77 ± 37.63	4.6	795.92 ± 12.83	1.6
Amygdalin	4	4.05 ± 0.29	7.0	4.43 ± 0.11	2.5	4.26 ± 0.51	12.1	4.23 ± 0.34	8.0
	40	38.56 ± 1.18	3.1	40.32 ± 1.56	3.9	41.96 ± 3.56	8.5	38.76 ± 3.80	9.8
	800	796.87 ± 32.71	4.1	802.72 ± 21.27	2.7	815.59 ± 8.14	1.0	785.65 ± 47.93	6.1
Maslinic acid	2	2.12 ± 0.26	12.5	1.97 ± 0.15	7.4	2.01 ± 0.13	6.4	2.03 ± 0.11	5.4
	20	19.57 ± 1.18	6.0	18.66 ± 0.59	3.1	19.48 ± 1.67	8.6	18.64 ± 1.75	9.4
	400	375.59 ± 40.00	10.7	391.56 ± 11.81	3.0	383.73 ± 22.51	5.9	384.32 ± 18.50	4.8
Corosolic acid	2	1.96 ± 0.13	6.7	1.98 ± 0.14	7.0	1.96 ± 0.07	3.5	2.10 ± 0.13	6.2
	20	20.42 ± 0.64	3.1	19.51 ± 0.30	1.6	18.54 ± 2.25	12.1	20.02 ± 0.58	2.9
	400	374.37 ± 42.55	11.4	407.67 ± 9.67	2.4	378.25 ± 19.65	5.2	386.56 ± 22.55	5.8
Rutin	1	1.03 ± 0.11	10.7	0.96 ± 0.11	11.1	1.04 ± 0.12	11.3	1.03 ± 0.12	11.8
	10	9.34 ± 0.75	8.0	9.60 ± 0.86	9.0	10.59 ± 0.74	7.0	9.45 ± 0.31	3.3
	200	190.37 ± 4.78	2.5	208.64 ± 6.54	3.1	217.66 ± 21.03	9.7	189.41 ± 9.00	4.8

### 3.4 Pharmacokinetic study

**FIGURE 3:**
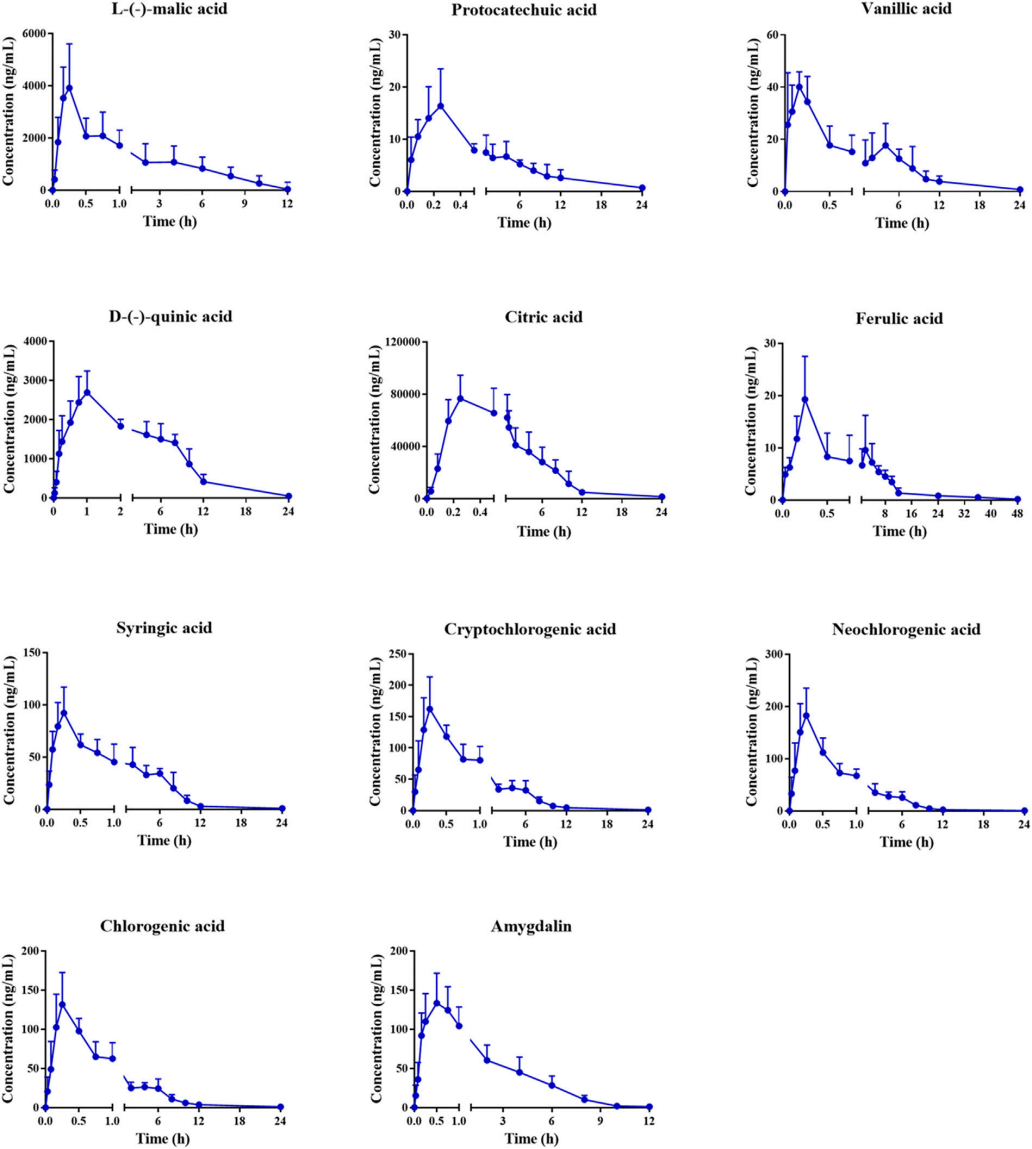
Mean plasma concentration–time profiles of L-(-)-malic acid, protocatechuic acid, vanillic acid, D-(-)-quinic acid, citric acid, ferulic acid, syringic acid, cryptochlorogenic acid, neochlorogenic acid, chlorogenic acid and amygdalin in rats after oral administration of MF extract.

The validated method was applied to the pharmacokinetic study of the 16 plasma components after intragastric administration of the MF extract. After oral administration of the MF extract, 3,4-dihydroxybenzaldehyde, caffeic acid, corosolic acid, maslinic acid, and rutin in rat plasma were only detected at the first few plasma sampling points, which made it difficult to obtain a complete pharmacokinetic profile. Therefore, we excluded these analytes from our results. The mean plasma concentration-time profiles of the other 11 components are shown in Figure 3, and the relevant pharmacokinetic parameters are summarised in Table 5.

**TABLE 5:** Pharmacokinetic parameters of 11 analytes in normal administration and model administration groups of rats. (Mean ± SD, *n* = 6).

Compound	T_max_	C_max_	T_1/2z_	AUC _(0-tn)_	AUC _(0-∞)_	MRT _(0-tn)_	MRT _(0-∞)_
	(h)	(ng/ml)	(h)	(ng/mL*h)	(ng/mL*h)	(h)	(h)
L-(-)-malic acid	0.22 ± 0.05	4,526.22 ± 1,453.89	3.09 ± 2.12	9925.14 ± 4,861.87	11435.37 ± 5,510.97	3.41 ± 0.38	4.98 ± 1.79
Protocatechuic acid	0.21 ± 0.07	17.10 ± 6.30	8.60 ± 4.92	93.55 ± 42.16	98.46 ± 49.90	9.21 ± 2.70	11.05 ± 4.78
Vanillic acid	0.15 ± 0.07	42.45 ± 12.05	8.56 ± 6.18	185.84 ± 51.28	194.64 ± 60.49	8.31 ± 3.30	10.41 ± 6.93
D-(-)-quinic acid	0.92 ± 0.13	2797.18 ± 478.49	5.04 ± 1.99	20261.31 ± 3953.37	20292.33 ± 3939.30	7.15 ± 0.69	7.23 ± 0.72
Citric acid	0.24 ± 0.04	76716.35 ± 17574.79	2.74 ± 1.60	363607.76 ± 117773.24	367433.90 ± 119346.53	4.71 ± 0.50	4.97 ± 0.60
Ferulic acid	0.19 ± 0.05	12.81 ± 3.15	8.51 ± 6.79	94.31 ± 21.10	98.60 ± 25.03	9.71 ± 2.05	11.94 ± 6.16
Syringic acid	0.28 ± 0.12	95.77 ± 20.12	10.95 ± 9.38	378.12 ± 46.20	384.31 ± 52.78	5.81 ± 1.60	6.76 ± 2.67
Cryptochlorogenic acid	0.29 ± 0.10	164.54 ± 48.02	9.16 ± 6.82	437.47 ± 120.93	450.02 ± 133.33	5.45 ± 1.53	6.88 ± 3.05
Neochlorogenic acid	0.28 ± 0.12	185.45 ± 49.95	8.14 ± 6.75	355.42 ± 85.06	359.03 ± 87.50	4.48 ± 1.60	5.08 ± 2.15
Chlorogenic acid	0.29 ± 0.10	134.90 ± 36.62	8.62 ± 5.61	331.45 ± 80.24	336.01 ± 76.04	5.70 ± 0.72	6.79 ± 2.68
Amygdalin	0.42 ± 0.13	137.89 ± 34.82	3.86 ± 1.18	450.79 ± 89.83	463.90 ± 95.05	4.64 ± 2.33	7.53 ± 5.07

As shown in Table 5, the T_max_ values of L-(-)-malic acid, protocatechuic acid, vanillic acid, D-(-)-quinic acid, citric acid, ferulic acid, syringic acid, cryptochlorogenic acid, neochlorogenic acid, chlorogenic acid, and amygdalin were 0.22, 0.21, 0.15, 0.92, 0.24, 0.19, 0.28, 0.29, 0.28, 0.29, and 0.42 h, respectively. These results indicate that the organic acids were rapidly absorbed in the body. The T_max_ values of cryptochlorogenic, neochlorogenic, and chlorogenic acids were similar to those previously reported (Shen et al., 2019; Sun et al., 2018). The T_1/2_ values of L-(-)-malic acid, citric acid, D-(-)-quinic acid, and amygdalin were 3.09, 2.74, 5.04, and 3.86 h, respectively, which revealed that these four analytes are eliminated shortly after oral administration. Similar pharmacokinetic trends for amygdalin have been reported in previous studies (Yang et al., 2021). The T_1/2_ values of protocatechuic, vanillic, ferulic, syringic, cryptochlorogenic, neochlorogenic, and chlorogenic acids were 8.60, 8.56, 8.51, 10.95, 9.16, 8.14, and 8.62 h, respectively, suggesting that these analytes are present in the body for a relatively long period and may exert a sustained therapeutic effect. The AUC_(0-tn)_ and AUC _(0-∞)_ values of L-(-)-malic, citric, and D-(-)-quinic acids were much higher than those of the other components, indicating that these three components had a higher level of plasma exposure, which was related to their high MF content. Moreover, the C_max_ values of L-(-)-malic and citric acids were 4,526.22 and 76716.35 ng/ml, respectively. Combined with their high extent of *in vivo* exposure, L-(-)-malic and citric acids could be the main effective substances in the MF extract, which is consistent with previous reports (Chen et al., 2006; Wang et al., 2010; Lin et al., 2013).

As shown in Figure 3, the concentration-time curves of ferulic and vanillic acids exhibited a double-peak phenomenon, which can be attributed to enterohepatic circulation (Wang et al., 2014). In addition, the duration of drug stay in the stomach and intestine largely depends on gastric emptying and gastrointestinal motility, which affect the rate and extent of drug absorption after oral administration. Compared with the small intestine, drugs are rarely absorbed in the stomach, as they are retained in the stomach until they are delivered to the small intestine and subsequently absorbed again, resulting in the double-peak phenomenon (Godfrey et al., 2011).

## 4 Conclusion

In this study, an UPLC-MS/MS method was established and validated for the simultaneous determination of the 16 components in rat plasma after oral administration of the MF extract. This method had excellent specificity, precision, recovery and stability. More importantly, the analytical method was the first pharmacokinetic study on the extract of MF, which provides valuable information for the development and clinical application of MF.

## Data Availability

The original contributions presented in the study are included in the article/Supplementary Material, further inquiries can be directed to the corresponding authors.

## Abbreviations

AUC, area-under-the-curve; Cmax, concentration maximum; IS, internal standard; LLOQ, lower limits of quantitation; MF, Prunus mume fructus; MRM, multiple reaction monitoring; MRT, mean residence time; QC, quality control; RE, relative error; RSD, relative standard deviation; S/N, signal-to-noise ratio; Tmax, concentration maximum; T1/2, plasma half-life; TCM, raditional Chinese medicine; UPLC-MS/MS, ultra-high performance liquid chromatography/tandem mass spectrometry.
