# Supplementary material for: Multi-component pharmacokinetic study of prunus mume fructus extract after oral administration in rats using UPLC-MS/MS
Source: Front Pharmacol. 2022 Sep 23;13:954692. doi: 10.3389/fphar.2022.954692 (PMC9541882; doi:10.3389/fphar.2022.954692)
Supplement: Supplementary file 1 [file DataSheet1.docx]

Supplementary Material

**Supplementary Table S1.** Mass spectrometry parameters of 16 analytes and IS in MF extract.

| Compound | Precursor Ion (*m/z*) | Product Ion (*m/z*) | Fragmentor (V) | Collision Energy (V) | Ion mode |
| --- | --- | --- | --- | --- | --- |
| L-(-)-malic acid | 133.0 | 115.1 | 70 | 8 | Negative |
| 3,4-Dihydroxybenzaldehyde | 137.0 | 108.0 | 118 | 28 | Negative |
| Protocatechuic acid | 153.0 | 109.0 | 98 | 16 | Negative |
| Vanillic acid | 167.0 | 152.0 | 93 | 12 | Negative |
| Caffeic acid | 179.0 | 135.0 | 88 | 16 | Negative |
| D-(-)-quinic acid | 191.0 | 93.0 | 136 | 24 | Negative |
| Citric acid | 191.0 | 111.0 | 80 | 8 | Negative |
| Ferulic acid | 193.1 | 134.0 | 93 | 16 | Negative |
| Syringic acid | 197.0 | 182.0 | 98 | 12 | Negative |
| Cryptochlorogenic acid | 353.1 | 173.1 | 113 | 16 | Negative |
| Neochlorogenic acid | 353.1 | 191.0 | 113 | 20 | Negative |
| Chlorogenic acid | 353.1 | 191.0 | 103 | 12 | Negative |
| Amygdalin | 456.1 | 323.1 | 176 | 8 | Negative |
| Maslinic acid | 471.4 | 471.4 | 275 | 39 | Negative |
| Corosolic acid | 471.4 | 471.4 | 305 | 5 | Negative |
| Rutin | 609.1 | 300.0 | 219 | 40 | Negative |
| Rosmarinic acid  (IS) | 359.0 | 161.0 | 128 | 16 | Negative |

**Supplementary Table S2.** The content of 16 analytes in MF extract (n = 3).

| Compound | Content (μg/g) |
| --- | --- |
| L-(-)-malic acid | 5105.05±210.22 |
| 3,4-Dihydroxybenzaldehyde | 37.46±3.04 |
| Protocatechuic acid | 144.73±4.57 |
| Vanillic acid | 131.16±77.97 |
| Caffeic acid | 29.00±17.56 |
| D-(-)-quinic acid | 7189.45±687.34 |
| Citric acid | 306440.80±21590.02 |
| Ferulic acid | 21.11±4.54 |
| Syringic acid | 113.36±10.59 |
| Cryptochlorogenic acid | 3371.60±288.33 |
| Neochlorogenic acid | 2615.07±321.92 |
| Chlorogenic acid | 2716.04±155.43 |
| Amygdalin | 1363.04±251.83 |
| Maslinic acid | 60.63±3.17 |
| Corosolic acid | 156.25±4.37 |
| Rutin | 13.60±1.67 |
